# Supplementary material for: End Sequence Analysis Toolkit (ESAT) expands the extractable information from single-cell RNA-seq data
Source: Genome Res. 2016 Oct;26(10):1397–410. doi: 10.1101/gr.207902.116 (PMC5052061; doi:10.1101/gr.207902.116)
Supplement: Supplemental Material [file supp_gr.207902.116_Supp_Materials.tar.gz › ESAT-esat_v0.1/doc/html/functions_func_c.html]

ESAT: Class Members - Functions


ESAT


- Main Page
- Related Pages
- Packages
- Classes
- Files

- Class List
- Class Index
- Class Hierarchy
- Class Members

- All
- Functions
- Variables
- Enumerator

- a
- b
- c
- d
- e
- f
- g
- h
- i
- j
- k
- l
- m
- n
- o
- p
- q
- r
- s
- t
- u
- v
- w
- z


All Classes Namespaces Files Functions Variables Enumerator Pages

### - c -

- calcAllTermsEnrichment()
  : broad.pda.gene.functionalAnnotation
- calcLightSilhouette()
  : broad.pda.geneexpression.clustering.Kmeans
- calcSilhouette()
  : broad.pda.geneexpression.clustering.Kmeans
- calcTermEnrichment()
  : broad.pda.gene.functionalAnnotation
- calculateApproximatePVal()
  : broad.core.math.ScanStatistics
- calculateBamEnrichments()
  : broad.pda.seq.rap.CollectAnnotationEnrichments
- calculateDiscreteAnnotationEnrichments()
  : broad.pda.seq.rap.CollectAnnotationEnrichments
- calculateGc()
  : broad.pda.seq.rap.GCContent
- calculateGlobalPvalue()
  : umms.core.model.score.BasicScanStatisticScore
- calculateLocalPvalue()
  : umms.core.model.score.BasicScanStatisticScore
- calculateNormalizationFactors()
  : umms.esat.ESAT
  , umms.esat.ESATDistanceCalculator
- calculatePVal()
  : broad.core.math.ScanStatistics
  , broad.pda.seq.segmentation.ContinuousDataAlignmentModel
  , umms.core.model.score.BinomialEnrichmentScore
  , umms.core.model.score.PoissonEnrichmentScore
- calculateScore()
  : jaligner.Alignment
- callKLPeaks()
  : broad.pda.seq.rap.RAPPeakCaller
- callSkellamPeaks()
  : broad.pda.seq.rap.RAPPeakCaller
- Cell()
  : jaligner.Cell
- centroidOf()
  : broad.core.motif.PositionWeightMatrix
- changeBackground()
  : broad.core.siphy.EvolutionaryModel
- checkOrientation()
  : broad.pda.seq.graph.ChromosomeWithBubblesJGraphT
- checkScore()
  : jaligner.Alignment
- chiSquareDistance()
  : broad.core.math.EmpiricalDistribution
- chol()
  : Jama.Matrix
- CholeskyDecomposition()
  : Jama.CholeskyDecomposition
- Chromosome()
  : broad.pda.feature.genome.Chromosome
- ChromosomeInconsistencyException()
  : umms.core.alignment.ChromosomeInconsistencyException
- chromosomeLength()
  : broad.pda.seq.segmentation.AlignmentDataModelStats
- ChromosomeWithBubblesJGraphT()
  : broad.pda.seq.graph.ChromosomeWithBubblesJGraphT
- chunk()
  : broad.core.sequence.Sequence
- CisVsTrans()
  : broad.pda.differentialExpression.CisVsTrans
- ClassifyAgilentLincProbes()
  : broad.pda.geneexpression.agilent.ClassifyAgilentLincProbes
- cleanIsoforms()
  : broad.pda.gene.GeneWithIsoforms
- cleanup()
  : broad.pda.seq.rap.CollectAnnotationEnrichments
- clear()
  : broad.core.annotation.AnnotationReader< T extends GenomicAnnotation >
  , broad.core.datastructures.IntervalTree< V >
  , broad.core.multiplealignment.MAFAlignment
  , broad.core.parser.StringParser
  , broad.core.siphy.TreeScalerIO
  , broad.pda.annotation.BEDFileParser
- clearAttribute()
  : broad.core.annotation.GFF
- clearFullIntervalTreeAsAlignmentsCached()
  : broad.pda.seq.segmentation.AlignmentDataModel
  , broad.pda.seq.segmentation.GenericAlignmentDataModel
- clone()
  : Jama.Matrix
- cloneRegions()
  : broad.core.annotation.AnnotationReader< T extends GenomicAnnotation >
- close()
  : broad.core.datastructures.JCSCache.JCSNodeIterator
  , broad.pda.samtools.PairedEndReadIterator
  , broad.pda.samtools.SAMPairedEndFileReader
  , broad.pda.samtools.WrappedIterator
  , broad.pda.seq.fastq.FastqParser
  , broad.pda.seq.segmentation.WrappedIGVIterator
  , umms.core.coordinatesystem.ShuffledIterator< T extends Annotation >
  , umms.core.general.CloseableFilterIterator< T >
  , umms.core.model.AlignmentModel.FilteredIterator
  , umms.core.model.AlignmentModel.NodeIterator
  , umms.core.model.AlignmentModel.WrapAlignmentCountIterator
  , umms.core.model.JCSAlignmentModel.FilteredIterator
  , umms.core.model.JCSAlignmentModel.NodeIterator
  , umms.core.model.JCSAlignmentModel.WrapAlignmentCountIterator
  , umms.core.model.score.WindowScoreIterator< T extends WindowScore >
  , umms.core.readers.PairedEndReader
  , umms.core.writers.PairedEndWriter
- CloseableFilterIterator()
  : umms.core.general.CloseableFilterIterator< T >
- closeFilterRejectWriters()
  : broad.pda.seq.protection.MultiSampleScanPeakCaller
- closeTo1()
  : broad.core.math.MathUtil
- CLUSTAL()
  : jaligner.formats.CLUSTAL
- Cluster()
  : broad.pda.geneexpression.clustering.Cluster
- cluster()
  : broad.pda.geneexpression.clustering.HierarchicalClustering
- CLUtil()
  : broad.core.util.CLUtil
- colapse()
  : broad.pda.gene.GeneWithIsoforms
- collapse()
  : umms.core.annotation.AbstractAnnotationCollection< T extends Annotation >
  , umms.core.annotation.AnnotationCollection< T extends Annotation >
  , umms.core.annotation.AnnotationList< T extends Annotation >
- collapseByIntersection()
  : broad.core.util.CollapseByIntersection
- CollapsedGeneLengths()
  : umms.esat.CollapsedGeneLengths
- CollapseGenesByIntersection()
  : broad.core.util.CollapseByIntersection
- collapseOverlappers()
  : umms.core.utils.AnnotationUtils
- CollapseProbesByMinPValue()
  : broad.pda.geneexpression.agilent.CollapseProbesByMinPValue
- CollapseProjections()
  : broad.pda.differentialExpression.CollapseProjections
- collapseSingleExonTranscripts()
  : broad.pda.annotation.BEDFileParser
- collectResults()
  : broad.pda.seq.rap.CollectAnnotationEnrichments
- columnDensityNormalization()
  : broad.core.datastructures.MatrixWithHeaders
- columnDimension()
  : broad.core.datastructures.MatrixWithHeaders
- CombinationGenerator()
  : broad.core.math.CombinationGenerator
- commandLineHasDebugFlag()
  : broad.pda.seq.protection.MultiSampleScanPeakCaller
- commandLineOutDir()
  : broad.pda.seq.protection.MultiSampleScanPeakCaller
- CommandLineParser()
  : broad.core.parser.CommandLineParser
- commaSeparatedStringToList()
  : broad.core.siphy.ConservationUtils
- compare()
  : broad.core.util.ChromosomeStringComparator
  , jaligner.matrix.MatricesCompartor
- compareNames()
  : broad.pda.feature.genome.Chromosome
- compareTo()
  : broad.core.annotation.BasicGenomicAnnotation
  , broad.core.annotation.BasicLightweightAnnotation
  , broad.core.primer3.HairpinKmer
  , broad.core.sequence.SequenceRegion
  , broad.pda.feature.genome.Chromosome
  , broad.pda.seq.graph.Path
  , umms.core.alignment.FeatureSequenceAlignment.UnorderedGenePair
  , umms.core.annotation.AbstractAnnotation
  , umms.core.annotation.CompoundInterval
  , umms.core.annotation.Gene
  , umms.core.annotation.SingleInterval
  , umms.core.feature.GeneWindow
  , umms.core.model.score.CountScore
  , umms.core.model.score.EndInWindowCountScore
  , umms.core.model.score.StartInWindowCountScore
- compareToAnnotation()
  : broad.core.sequence.SequenceRegion
  , umms.core.annotation.AbstractAnnotation
  , umms.core.annotation.Annotation
- compatible()
  : umms.core.scripture.BuildScriptureCoordinateSpace
- complement()
  : broad.core.primer3.ComputeMIRScore
  , broad.core.sequence.Sequence
  , broad.core.sequence.SequenceRegion
  , umms.core.annotation.Annotation
  , umms.core.annotation.BasicAnnotation
  , umms.core.annotation.CompoundInterval
- CompoundInterval()
  : umms.core.annotation.CompoundInterval
- compress()
  : broad.core.multiplealignment.MAFAlignment
  , broad.core.multiplealignment.MultipleAlignment
- compressMatrixByRowDescriptions()
  : broad.core.datastructures.MatrixWithHeaders
- compute454Flow()
  : broad.core.sequence.Sequence
- computeAbsFoldMatrix()
  : broad.pda.differentialExpression.DifferentialScoring
- ComputeConservedMotifs()
  : broad.core.motif.misc.ComputeConservedMotifs
- ComputeCorrelationOfReplicates()
  : broad.pda.geneexpression.clustering.ComputeCorrelationOfReplicates
- ComputeEnrichments()
  : broad.pda.seq.chip.ComputeEnrichments
- computeFoldMatrix()
  : broad.pda.differentialExpression.DifferentialScoring
- computeGCContent()
  : broad.core.sequence.Sequence
- computeGCPercent()
  : broad.core.primer3.ComputeOriginalScore
- computeGlobalStats()
  : umms.core.model.AlignmentModel
  , umms.core.model.JCSAlignmentModel
- ComputeHypergeomtricEnrichment()
  : broad.pda.geneexpression.ComputeHypergeomtricEnrichment
- computeJMatrix()
  : broad.core.siphy.EvolutionaryModel
- ComputeKmersForRegion()
  : broad.core.siphy.tools.conservation.ComputeKmersForRegion
- computeLikelihood()
  : broad.core.siphy.EvolutionaryModel
- ComputeMaxPercentileOfElements()
  : broad.core.siphy.tools.conservation.ComputeMaxPercentileOfElements
- computeMaxTM()
  : broad.core.primer3.PrimerUtils
- computeMIRScore()
  : broad.core.primer3.ComputeMIRScore
- computeOriginalScore()
  : broad.core.primer3.ComputeOriginalScore
- computeOriginalScorePerKMer()
  : broad.core.primer3.ComputeOriginalScore
- computePathLogLikelihood()
  : broad.core.hmm.MarkovModel< T >
  , broad.core.siphy.PiHMM
- ComputePercentageOfKmers()
  : broad.core.siphy.tools.conservation.ComputePercentageOfKmers
- computePermutedZScores()
  : broad.core.math.Statistics
- computeProb()
  : broad.core.motif.PositionWeightColumn
- computeScore()
  : broad.core.sequence.SequenceRegionWindow
- computeScoreDistribution()
  : broad.core.motif.PositionWeightMatrix
- computeSequenceBGSequences()
  : broad.core.motif.PWMUtils
- computeSequenceComposition()
  : broad.core.sequence.Sequence
- computeTestStatistics()
  : broad.pda.differentialExpression.DifferentialScoring
- computeTM()
  : broad.core.primer3.PrimerUtils
- computeTM2()
  : broad.core.primer3.PrimerUtils
- computeTransitions()
  : broad.core.siphy.EvolutionaryModel
- concatAnnotationSet()
  : broad.core.annotation.AnnotationReader< T extends GenomicAnnotation >
- cond()
  : Jama.Matrix
  , Jama.SingularValueDecomposition
- confidenceByColumn()
  : broad.core.datastructures.MatrixWithHeaders
- ConnectDisconnectedTranscripts()
  : umms.core.scripture.statistics.ConnectDisconnectedTranscripts
- connectVertexToGraph()
  : umms.core.scripture.OrientedChromosomeTranscriptGraph
- ConservedPWMScanner()
  : broad.core.siphy.ConservedPWMScanner
- constituentIntrons()
  : broad.pda.gene.GeneWithIsoforms
- constituentIsoform()
  : broad.pda.gene.GeneWithIsoforms
- constructWithCopy()
  : Jama.Matrix
- containChr()
  : broad.pda.annotation.BEDFileParser
- containedWithin()
  : broad.pda.datastructures.Alignments
- contains()
  : broad.core.annotation.BasicLightweightAnnotation
  , broad.core.annotation.LightweightGenomicAnnotation
  , broad.core.multiplealignment.MAFAlignment
  , broad.core.sequence.Sequence
  , broad.core.sequence.SequenceRegion
  , broad.pda.annotation.BEDFileParser
  , umms.core.annotation.Annotation
  , umms.core.annotation.BasicAnnotation
  , umms.core.annotation.CompoundInterval
  , umms.core.annotation.SingleInterval
- containsAnnotation()
  : broad.core.annotation.AnnotationReader< T extends GenomicAnnotation >
- containsAttribute()
  : umms.core.annotation.Gene
- containsColumn()
  : broad.core.datastructures.MatrixWithHeaders
- containsDataFor()
  : umms.core.coordinatesystem.GenomicSpace
  , umms.core.coordinatesystem.TranscriptInGenomicSpace
- containsExactInterval()
  : umms.core.annotation.CompoundInterval
- containsFullLengthUngappedMatch()
  : umms.core.alignment.SmithWatermanAlignment
- containsInterval()
  : umms.core.annotation.CompoundInterval
- containsReference()
  : umms.core.model.AlignmentModel
  , umms.core.model.JCSAlignmentModel
- containsRow()
  : broad.core.datastructures.MatrixWithHeaders
- contiguousEndSubSequenceOverMin()
  : broad.core.annotation.MaximumContiguousSubsequence
- contiguousStartSubSequenceOverMin()
  : broad.core.annotation.MaximumContiguousSubsequence
- ContinuousDataAlignmentModel()
  : broad.pda.seq.segmentation.ContinuousDataAlignmentModel
- convertInputToPairedEnd()
  : umms.core.writers.PairedEndWriter
- ConvertToDiscreteMatrix()
  : broad.pda.differentialExpression.ConvertToDiscreteMatrix
- convertToFasta()
  : broad.pda.seq.fastq.FastqParser
- convertToNumberedFasta()
  : broad.pda.seq.fastq.FastqParser
- convertToPairedEndFragment()
  : broad.pda.samtools.SAMPairedEndFileReader
- copy()
  : broad.core.datastructures.MatrixWithHeaders
  , broad.core.motif.PositionWeightMatrix
  , broad.core.sequence.SequenceRegion
  , broad.core.siphy.EvolutionaryModel
  , broad.core.siphy.EvolutionaryModelParameters
  , broad.pda.annotation.BEDFileParser
  , broad.pda.seq.graph.Path
  , jaligner.Alignment
  , Jama.Matrix
  , umms.core.annotation.Annotation
  , umms.core.annotation.BasicAnnotation
  , umms.core.annotation.Gene
- CorrelationAgilentNanostring()
  : broad.pda.geneexpression.agilent.CorrelationAgilentNanostring
- CorrelationBetweenReplicates()
  : broad.pda.geneexpression.agilent.CorrelationBetweenReplicates
- CorrelationMatrix()
  : broad.pda.geneexpression.clustering.CorrelationMatrix
- count()
  : broad.pda.seq.segmentation.AlignmentDataModelStats
  , broad.pda.seq.segmentation.ContinuousDataAlignmentModel
- countLessThanLambda()
  : broad.pda.seq.rap.SkellamScore
- CountNums()
  : broad.pda.geneexpression.CountNums
- countOverlappers()
  : broad.core.overlaputils.GeneSetIntersect
- countReads()
  : broad.pda.seq.segmentation.GenericAlignmentDataModel
- CountScore()
  : umms.core.model.score.CountScore
- countSoftMaskedBases()
  : broad.core.sequence.Sequence
- countWithinExon()
  : broad.pda.seq.segmentation.AlignmentDataModelStats
- covariance()
  : broad.core.math.Statistics
- CoverageScore()
  : umms.core.model.score.CoverageScore
- create()
  : broad.core.annotation.AnnotationFactory< T extends LightweightGenomicAnnotation >
  , broad.core.annotation.AnnotationReaderFactory
  , broad.core.multiplealignment.MultipleAlignmentFactory
  , broad.core.multiplealignment.MultipleAlignmentIOFactory
  , umms.core.general.TabbedReader.Factory< T >
- createAnnotation()
  : broad.core.annotation.AnnotationReader< T extends GenomicAnnotation >
  , broad.core.annotation.BasicAnnotationReader
  , broad.core.annotation.BasicGenomicAnnotationReader
  , broad.core.annotation.BEDGraphReader
  , broad.core.annotation.BEDReader
  , broad.core.annotation.GFFReader
  , broad.core.annotation.RepeatMaskerAnnotationReader
  , broad.core.annotation.ShortBEDReader
  , broad.pda.datastructures.AlignmentsReader
- createAsciiFromBinary()
  : broad.core.sequence.FastaQualityBinaryIO
- createBinaryFromAscii()
  : broad.core.sequence.FastaQualityBinaryIO
- createDefaultTwoStateChain()
  : broad.core.siphy.PiHMM
- createEdge()
  : broad.pda.seq.graph.ChromosomeWithBubblesJGraphT
- createFromCommandArgs()
  : broad.pda.seq.protection.MultiSampleScanPeakCaller
- createFromUCSC()
  : broad.core.annotation.BasicLightweightAnnotation
  , umms.core.annotation.BasicAnnotation
- createIndex()
  : broad.core.multiplealignment.MAFAlignment
- createIndexFromQualityFile()
  : broad.core.sequence.FastaQualityIndex
- createIsoPWM()
  : broad.core.motif.PositionWeightMatrix
- createSizeFile()
  : broad.core.sequence.FastaSequenceIO
- createTwoStateChain()
  : broad.core.siphy.PiHMM
- createUnloadedAlignment()
  : broad.core.multiplealignment.MAFIO
- CrossRandModelsWithBed()
  : broad.pda.gene.transcriptsNullModel
- customCommandLineValidation()
  : broad.pda.samtools.PairedEndTDF
  , broad.pda.seq.rap.CollectAnnotationEnrichments
  , broad.pda.seq.rap.GenomeCommandLineProgram
  , broad.pda.seq.rap.GenomeScoringProgram
  , broad.pda.seq.rap.rna.TranscriptomeCommandLineProgram
  , broad.pda.seq.rap.rna.TranscriptomeScoringProgram
- CV()
  : broad.pda.seq.segmentation.ContinuousDataAlignmentModel


---

Generated on Fri Aug 15 2014 15:11:16 for ESAT by  

 1.8.7
